# Multi-omic analysis identifies a multi-step pathology in a case of multiple chorangioma syndrome in monochorionic twins

**DOI:** 10.1186/s13023-026-04228-2

**Published:** 2026-02-03

**Authors:** Brandon M. Wilk, Manavalan Gajapathy, Donna M. Brown, Virginia E. Duncan, Elizabeth A. Worthey

**Affiliations:** 1 Center for Computational Genomics and Data Science, Departments of Genetics, University of Alabama at Birmingham, Birmingham, AL USA; 2 Department of Pathology, Perinatal Section, University of Alabama at Birmingham, Birmingham, AL USA

**Keywords:** Placenta, Multiple chorangioma syndrome, Hypoxia, Genomics, Transcriptomics, EPAS1, COL1A1, FBXO11, TRIM71

## Abstract

**Background:**

Chorangiomas, benign capillary lesions of the placenta, occur in ~1% of births, typically as solitary nodules. Multiple chorangioma syndrome is rare and increases risk of fetal heart failure, hydrops fetalis, and intrauterine death due to placental hemodynamic disruption. While genetic and hypoxic factors have been suggested in chorangioma development, direct molecular evidence is lacking. Here, we present a unique case of multiple chorangiomas confined to half of a shared placenta in monozygotic monochorionic diamniotic twins, providing a rare opportunity to dissect molecular drivers of chorangioma formation.

**Methods:**

Formalin-fixed paraffin-embedded tissue samples from chorangioma, affected and unaffected villi, and decidua from MCDA twins were subjected to whole-genome and bulk RNA sequencing. Germline and somatic small, structural, and copy number variants were identified. Clonal evolution analysis was conducted using PyClone-VI and ClonEvol. Mutational signature profiling was performed with SigProfilerExtractor to characterize molecular drivers of pathology. RNA-Seq (100 M reads/sample) was processed using the nf-core RNA-Seq pipeline, with differential expression analysis via DESeq2 to identify transcriptomic changes and mutational signatures. Quality control and artifact filtering were applied to mitigate FFPE-induced sequencing errors.

**Results:**

We identified a likely pathogenic early embryonic or germline EPAS1 frameshift deletion, suggesting impaired placental oxygen regulation and VEGF-driven angiogenesis. Chorangioma-affected tissue harbored pathogenic COL1A1, FBXO11, and TRIM71 somatic mutations, with elevated Leptin expression and oxidative stress signatures. In contrast, the unaffected twin’s placental territory carried a pathogenic MUTYH variant and repair deficiency-associated mutational signatures. These findings reveal distinct molecular processes in each placental domain and suggest a predisposing genetic alteration.

**Conclusions:**

Our study provides novel insights into the molecular basis of multiple chorangioma syndrome. To our knowledge, it is the first study to propose a molecular mechanism and the first to propose interaction of germline and somatic variants in syndrome pathobiology. Identification of molecular signatures linked to malignancy suggests potential overlap with oncogenic pathways that, if confirmed, would extend understanding of placental biology. These findings highlight the genetic-environmental interplay in placental pathology, with implications for preeclampsia, intrauterine growth restriction, and fetal vascular malperfusion.

**Supplementary Information:**

The online version contains supplementary material available at 10.1186/s13023-026-04228-2.

## Background

Chorangiomas (chorioangiomas) are common, non-cancerous vascular tumors of the placenta, that occur in about 1% of births [1, 2]. When present as solitary nodules, they are considered not to be of clinical significance [1, 2]. In rare situations, where they are large (> 4 cm in diameter) or occuring as multiple chorangiomas (occupying > 80% of the placental parenchyma), they greatly increase the risks of polyhydramnios, heart failure from arteriovenous shunting, growth restriction, hydrops fetalis, preterm delivery, sudden intrauterine fetal death, and/or stillbirth of the fetus, as well as risks of preeclampsia and HELLP (Hemolysis, Elevated Liver enzymes and Low Platelets) syndrome in the mother [1, 3, 4]. A recent review of multiple chorangioma cases found that only 4 of 11 affected babies survived [3]. Chorangiomas are often identified in the second trimester or later [5], but may be discovered incidentally due to other complications or after birth [6].

The etiology of chorangioma formation remains unclear, though genetic and environmental factors likely play roles [7, 8]. Chorangiomas and related hamartomas, such as placental mesenchymal dysplasia, occur in association with Beckwith-Wiedemann syndrome (BWS), a rare genetic disorder characterized by overgrowth and an increased risk of certain childhood cancers, although the genetic basis of this connection is unclear [7, 9–13]. Cases of familial chorangiomas and instances of recurrent multiple chorangiomas suggest a possible genetic predisposition, but evidence is limited due to the low case volume [3, 14, 15]. Environmental factors, particularly hypobaric hypoxia, have been linked to chorangioma formation [13]. Chorangiomas occur more frequently in native populations living at high altitudes exposed to chronic hypoxia [8, 16], as well as in less perfused regions of the placenta [9]. Aberrant angiogenesis driven by hypoxia-induced vascular endothelial growth factor (VEGF) signaling and altered expression of angiogenic factors have been proposed as mechanisms [17]. Increased chorangioma incidence has been noted in some twin and multiple pregnancies [18, 19], although the mechanism of this association are not known [17, 18, 20].

We present a case of monozygotic, monochorionic diamniotic (MCDA) twins sharing a placenta, with extensive multiple chorangiomas confined to a single baby’s placental territory. The rarity of multiple chorangioma syndrome and the unique presentation in MCDA twins sharing a single placenta provided an ideal opportunity to investigate potential molecular underpinnings of chorangioma formation. This unique case enabled us to investigate molecular factors in such placental outcomes. To determine why only one twin’s placental territory was affected, we performed whole-genome and transcriptome sequencing of chorangioma and placental tissues, identifying distinct molecular variations, mutational signatures, and expression changes. This case offers novel insights into chorangioma development.

## Methods

### Sample preparation

**Fig. 1:**
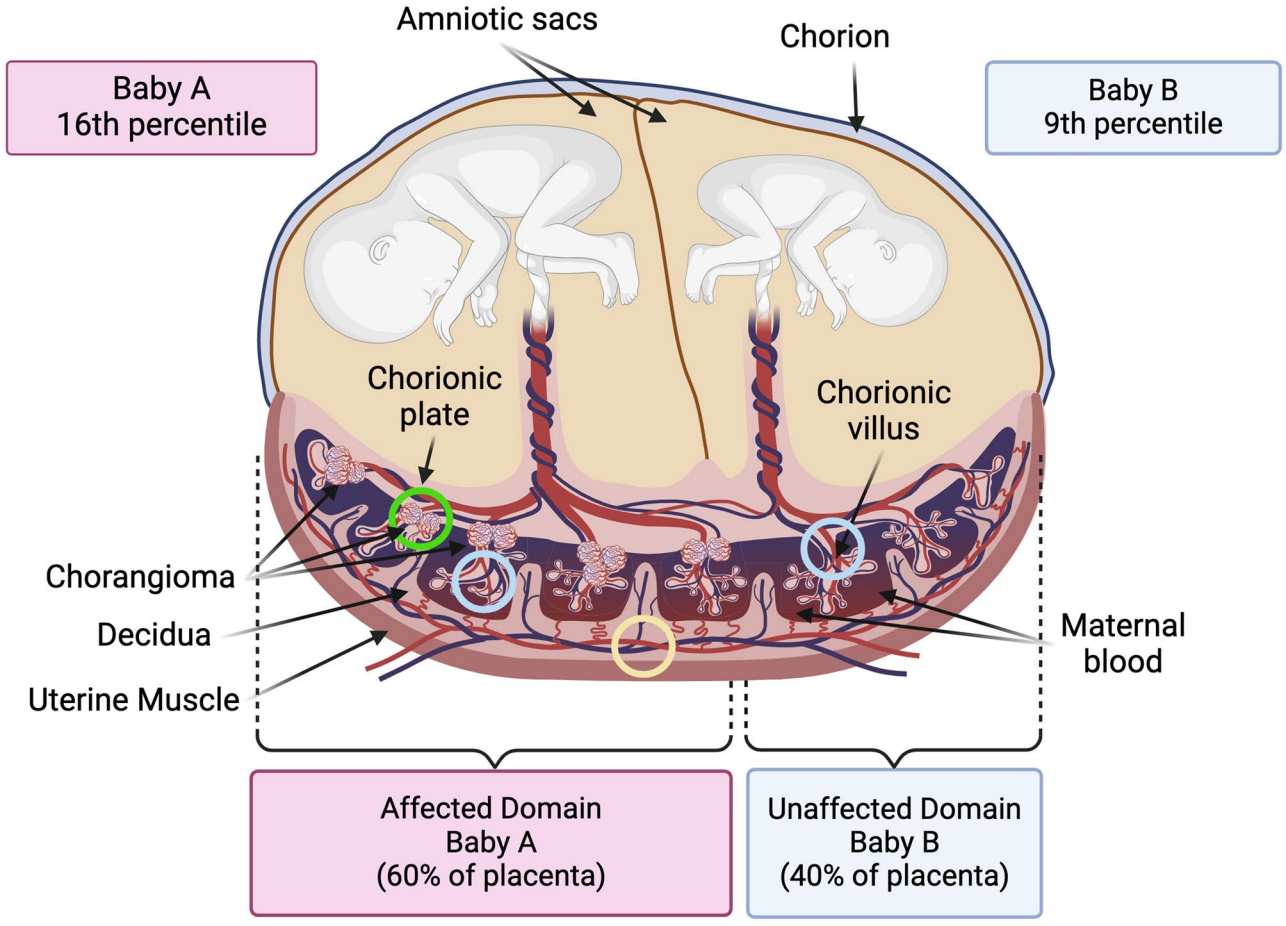
Twin and placental findings. Birth weight percentiles for each twin are shown. The placental chorionic vasculature (referred to as domains) is shown for both babies. Baby A’s domain contained an abundance of chorangiomas “affected domain”, whilst baby B’s domain was free of chorangiomas “unaffected domain”. The location of the samples gathered for omic analyses are circled. Green denotes affected domain chorangioma tissue, light blue denotes unaffected villus tissue in each domain. Yellow denotes decidua tissue at the maternal surface

Pathologic evaluation of the placenta was performed according to standard institutional protocols based on Amsterdam Criteria [21]. Formalin-fixed, paraffin-embedded (FFPE) blocks and H&E stained slides were prepared according to routine laboratory protocols. Chorangiomas exhibited extensive involvement, confined to baby A’s placental territory, with minimal identifiable normal placental tissue (Fig. 1). Regions of interest were identified on H&E (hematoxylin and eosin) stained slides and 2-mm core punches from unaffected villi, chorangioma tissue, and decidua from baby A’s placental territory and from normal villi from baby B’s placental territory were collected for sequencing (Fig. 1). These were sent to the Vanderbilt University Medical Center (VUMC) VANTAGE lab for DNA and RNA isolation (the unaffected villi from baby A’s placental territory only had RNA isolated) and sequencing.

## Whole genome sequencing and analysis

DNA was isolated using Qiasymphony and/or FlexSTAR+ and quantified via Qubit and agarose gel electrophoresis to ensure sufficient yield and quality. Libraries were prepared with the Twist Biosciences kit (P/N: 104,207) following manufacturer’s protocols. Library quality was assessed using the Agilent Bioanalyzer and quantified with qPCR using the KAPA Library Quantification Kit (P/N: KK4873) and QuantStudio 12K instrument. Library pools were combined in equimolar ratios, and cluster generation performed on a NovaSeq 6000 System. 150 bp paired-end sequencing targeted 400 M reads per sample. Sequencing FASTQ files underwent QC, including read quality assessment, using Real Time Analysis Software and NovaSeq Control Software (v1.8.0), with MultiQC (v1.7) for data quality checks. Due to DNA degradation from fixation, each sample went through two library preparation and sequencing workflows to achieve sufficient mean coverage (> 30x). This threshold was selected based on the hypothesis that, given the extensive placental involvement (> 90% of total parenchymal volume), formation resulted from an early embryonic variant. Variants present at variant allele fractions of ≥25%, as presumed, are reliably detectable at this coverage with ≥99% probability [22, 23].

Reads were downloaded to UAB’s Cheaha supercomputer and aligned to the human GRCh38 reference genome using BWA-MEM (v0.7.15) [24]. Small, structural (SV), and copy number (CNV) variants were called following GATK best practices [25] using haplotypecaller (v4.0.5.1) for germline variants, Mutect2 (v2.2) for somatic variants [26], and Manta (v1.6.0) for SVs and CNVs [27]. Somatic variants were called from chorangioma and villus tissue in tumor + normal mode, with the maternal surface (decidua) sample as the normal. Quality control (QC) for sequencing data, alignment, and variant calling was performed using QuaC (v1.0) [28]. All samples met threshold for analysis (mean coverage for chorangioma was 34x, normal villi 38x, and maternal surface 57x). Chorangioma and normal villi had a high degree of unalignable reads (36.4% and 23.5%).

FFPE fixation can induce DNA variation resulting in false variant calls [29, 30]. FFPE induced variants were identified and removed using FFPE somatic error detection methods as previously described [31] using the Ideafix detection tool [32]. Verification of FFPE-induced variant removal was performed using FFPESig downloaded on December 11th, 2023 [33]. Variant annotation, filtering, and analysis were performed using Codicem [34], VarSome [35], and cBioPortal [36–38] following best practices for variant analysis [39]. In brief, filtering was performed for population allele frequencies (e.g. gnomAD [40]), in silico deleteriousness scores (e.g. CADD [41], PolyPhen-2 [42], and SIFT [43]), and gene–phenotype associations relevant to the phenotype of interest (e.g. ClinVar [44] and Human Phenotype Ontology (HPO) [45]). Variant analysis was performed to identify germline/early placental variation and variation unique to the chorangioma. Germline variant pathogenicity was classified according to American College of Medical Genetics and Genomics (ACMG) guidelines [46] and somatic variant pathogenicity according to Association for Molecular Pathology (AMP) guidelines [47].

## Mutational signature profiling

To assess mutational processes in the chorangioma tissue, we profiled somatic variant calls from both chorangioma and normal villus tissue [48]. We used SigProfilerExtractor v1.1.21 [49] to extract and deconstruct the mutational signatures comparing them to the 96 reference Single base substitutions (SBS) contexts from COSMIC’s Mutational Signature catalog v3.3 [50]. The tool was run with default parameters, using GRCh38 as the reference genome, with minimum_signatures set to 1 and maximum_signatures set to 10.

## Chorangioma subclone analysis

Germline bi-allelic single nucleotide variants (SNVs) from WGS data were analyzed with CNVKit [51] for allele-aware CNV calling and copy number estimates. Somatic bi-allelic SNVs were filtered for a minimum coverage of 35x and variant allele fraction (VAF) greater than or equal to 0.08 and combined with genome-wide copy number estimates. These were then used for clone clustering and clonal population fraction estimation using PyClone-VI v0.1.3 [52]. PyClone-VI was run twice, using binomial and beta-binomial distributions, with the latter selected for further analysis based on recommendations for over-dispersed WGS data [53]. ClonEvol v0.99.11 [54] was used to predict clonal evolution based on PyClone-VI clustering. Settings for ClonEvol are detailed in the clonal-evo.Rmd document in the clonal evolution code repository (data and materials).

## RNA sequencing analysis

RNA was isolated from tissue sites as highlighted in Fig. 1 and QC performed using Qubit or Picogreen with integrity assessed via BioAnalyzer or TapeStation. Library prep used NEBNext Ultra II Prep kits. Sequencing was conducted at VANTAGE on an Illumina NovaSeq 6000, generating paired end 150 bp reads, aiming for an average of 100 million reads per sample. Demultiplexed paired FASTQ outputs were returned. Initial QC was performed using FastQC, with adapter trimming and quality filtering via TrimGalore. QC metrics were poor due to RNA degradation, a known limitation with FFPE fixation [55, 56]. Given the extreme rarity of MCDA and multiple chorangioma events, advances in RNA sequencing from FFPE samples [57] and analysis improvements [58–60], we proceeded cautiously, making use of the nf-core RNA-Seq pipeline v3.6 [61] for alignment and expression quantification, with DESeq2 v1.28.0 for differential gene expression analysis.

## Results

### Case description

MCDA twins were delivered without complications at 35 weeks via Cesarean section (C-section). Antenatal scans showed mild intrauterine growth restriction (IUGR) of Baby B (9th percentile), while the growth of baby A was at the 16th percentile. Although there were no signs of Twin-to-Twin Transfusion Syndrome (TTTS) C-section was performed due to non-reassuring fetal status in twin B. The babies both had Apgar scores of 8 and 9 and did well in the initial postnatal period. The mother had three prior pregnancies, including one loss, for which we had no details. Pathologic examination of the shared placenta showed unequal placental territories (baby A: 60%, baby B: 40%). Overall placental weight was below the 5^th^ percentile for twin gestation (490 g; 558-971 g expected for 35 weeks). Multiple chorangiomas ranging in size from 0.1–1.5 cm in diameter were found extensively throughout the placental parenchyma (comprising > 90% of the total parenchymal volume), entirely confined to the placental territory of baby A (Fig. 1). WGS confirmed the twins were monozygotic, with both twins originating from a single zygote and sharing an identical genome.

## Germline variation in chorangioma formation

SV and CNV analysis revealed no variants of note, consistent with prior studies [20]. A novel likely pathogenic heterozygous near-splice 5-base frameshift deletion (c.22_26del, p.Lys8GlufsTer2) in Endothelial PAS Domain Protein 1 (EPAS1) was identified in the chorangioma tissue, decidua from baby A’s placental territory, and normal villi from baby B’s placental territory, indicating an early embryonic or germline origin (Table 1). RNA-Seq data confirmed expression of both wildtype and frameshift *EPAS1* alleles (Supplemental Fig. 1).

**Table 1:** Somatic and germline variants of interest

Tissue	Allele Origin	Gene	Variant	Classification	VAF*	GnomAD AF
All	Germline or Early Embryonic	EPAS1	NM_001430.5: c.22_26del (p.K8Efs*2)	Likely Pathogenic	51/52 /39	Not seen
Unaffected Villi	Somatic	MUTYH	NM_001048174.2: c.650 G > A (p.R217H)	Pathogenic/ Tier II	7.4	0.000024
Chorangioma	Somatic	COL1A1	NM_000088.4: c.1588 G > A (p.G530S)	Pathogenic/ Tier II	8.3	Not seen
Chorangioma	Somatic	FBXO11	NM_001190274.2: c.856 G > T (p.G286*)	Likely Pathogenic/ Tier II	8.6	Not seen
Chorangioma	Somatic	TRIM71	NM_001039111.3: c.2386C > T (p.R796C)	Likely Pathogenic/ Tier II	11	Not seen

Table 1 Legend: Classification of variants of interest based on ACMG and AMP guidelines. * VAF provided for EPAS1 within Chorangiomas, normal villi from baby B’s placental territory, and decidua. Germline and somatic variation was identified with somatic variation differing between affected and unaffected samples.

*EPAS1*, also known as Hypoxia-Inducible Factor 2 Alpha (HIF2A), is highly expressed in lung, adipose, and placental tissues, playing a central role in hypoxia response by regulating genes involved in metabolism, proliferation, angiogenesis, erythropoiesis, and VEGF expression [62–64]. *EPAS1* plays a crucial role in placental structure, angiogenesis, and embryonic development, supporting blood vessel and lung tubular system formation, particularly under hypoxic conditions [65–68]. Germline gain-of-function variants in *EPAS1* cause dominant familial erythrocytosis 4, marked by erythrocytosis and deep venous thrombosis [69]. Germline, somatic, or postzygotic gain-of-function *EPAS1* variants in early embryogenesis are also associated with congenital polycythemia, somatostatinoma, pheochromocytomas and paragangliomas syndrome, and Pacak–Zhuang syndrome [70–73]. Altered *EPAS1* expression has been linked to PI3K/mTORC2 activity, tumor angiogenesis, and malignant tumor progression [74–77]. In Tibetan populations, *EPAS1* variants have been shown to reduce expression in placental tissue under high-altitude hypoxia, blunting the physiological response to chronic hypoxia and coinciding with an increased incidence of chorangioma formation [16, 78–82].

Homozygous EPAS1 knockout in mice is lethal post-vasculogenesis due to improper blood vessel fusion in the yolk sac and embryo, and failure to form larger vessels [83, 84]. Heterozygous mice are viable but show blunted responses to chronic hypoxia [83, 85]. EPAS1 also directly regulates DNMT1 in lung tissue, linking it to DNA methylation defects and imprinting issues associated with about 50% of Beckwith-Wiedemann syndrome cases [86, 87].

## Somatic variation

No somatic CNVs or SVs of interest were identified. A pathogenic somatic variant (c.650 G > A, p.R217H) in *MUTYH* was identified in normal villi (Table 1). Expression could not be confirmed in RNA-Seq data due to insufficient coverage of this locus. This gene encodes a glycosylase involved in base excision repair (BER) of oxidative DNA damage, specifically removing adenine mispaired with 8-oxoguanine [88, 89]. The variant lies adjacent to the endonuclease active site, essential for repair initiation. When found constitutively in a homozygous or compound heterozygous state, this variant causes familial adenomatous polyposis and other cancer syndromes [88, 90]. This variant has also been identified as a somatic variant in malignant tumors [91].

Within the chorangioma, three somatic variants of interest were identified (Table 1). Their expression could not be confirmed in the RNA-Seq data due to insufficient gene coverage or absence of the allele. A pathogenic variant (c.1588 G > A, p.G530S) was identified in *COL1A1,* encoding the alpha-1 chain of type I collagen, essential for tensile strength and stability in connective tissues. Pathogenic germline variants in *COL1A1* cause autosomal dominant osteogenesis imperfecta and Ehlers-Danlos syndrome [92–94]. Somatic variants in *COL1A1* influence tumor extracellular matrix, affecting tissue stiffness and tumor progression [95]. In the placenta, *COL1A1* is essential for structural integrity, guiding cellular behaviors, activating PI3K-AKT signaling, epithelial-to-mesenchymal transition, angiogenesis, and hypoxia response [96, 97]. Disruptions in these processes can lead to vascular and placental abnormalities [98–100].

A likely pathogenic truncating variant (c.856 G > T, p.G286Ter) was identified in *FBXO11*, an E3 ubiquitin ligase essential for tagging proteins for degradation to maintain cellular protein balance [101–103]. Pathogenic germline variants in FBX011 cause autosomal dominant Intellectual developmental disorder with dysmorphic facies and behavioral abnormalities [104]. Dysregulation of *FBXO11* is linked to cancer and is often inactivated in lymphoma and other tumors, contributing to abnormal growth and tumor development [105, 106].

An additional likely pathogenic missense variant (c.2386C > T, p.R796C) was identified in *TRIM71*, an E3 ubiquitin ligase that regulates the cell cycle via RNA binding to the 3’ UTR of *CDKN1A/p21*, promoting NMD and embryonic stem cell proliferation [107, 108]. *TRIM71* is highly expressed in extravillous trophoblasts (EVTs) in the placenta [109–111]. Pathogenic germline variants in *TRIM71* are associated with autosomal dominant congenital hydrocephalus, characterized by cerebral ventriculomegaly, and altered expression has been linked to tumorigenesis [108, 112–114].

## Differential expression

Differential expression analysis was complicated by the tissue’s prolonged FFPE preservation. A 3.1-fold increase (p adj. < 0.05) in Leptin (*LEP*) expression was noted in chorangioma tissue compared to normal villous tissue. Leptin, a multifunctional endocrine protein, regulates angiogenesis, PI3K-AKT, and VEGF pathways in various cell types, including cancers [115]. A rare somatic SNV (chr7:g.128236766A > T) in an enhancer of *LEP* was found in the chorangioma tissue, though its effect is unclear. No significant expression differences were observed for the WGS-identified variants (Table 1). The proportion of pathogenic DNA variants leading to measurable expression changes varies considerably, influenced by variant type, genomic location, detection sensitivity, and biological context. In this case, although nonsense variants often trigger nonsense-mediated decay, pathogenic EPAS1 and FBXO11 variants have been identified with no measurable expression changes, instead manifesting through changes in protein stability or localization. Most missense variants leave expression intact, and in TRIM71, MUTYH, and COL1A1 specifically, pathogenic variants have been shown to disrupt protein structure and/or function leading to disease without alteration expression.

## Chorangioma clonal evolution

**Fig. 2:**
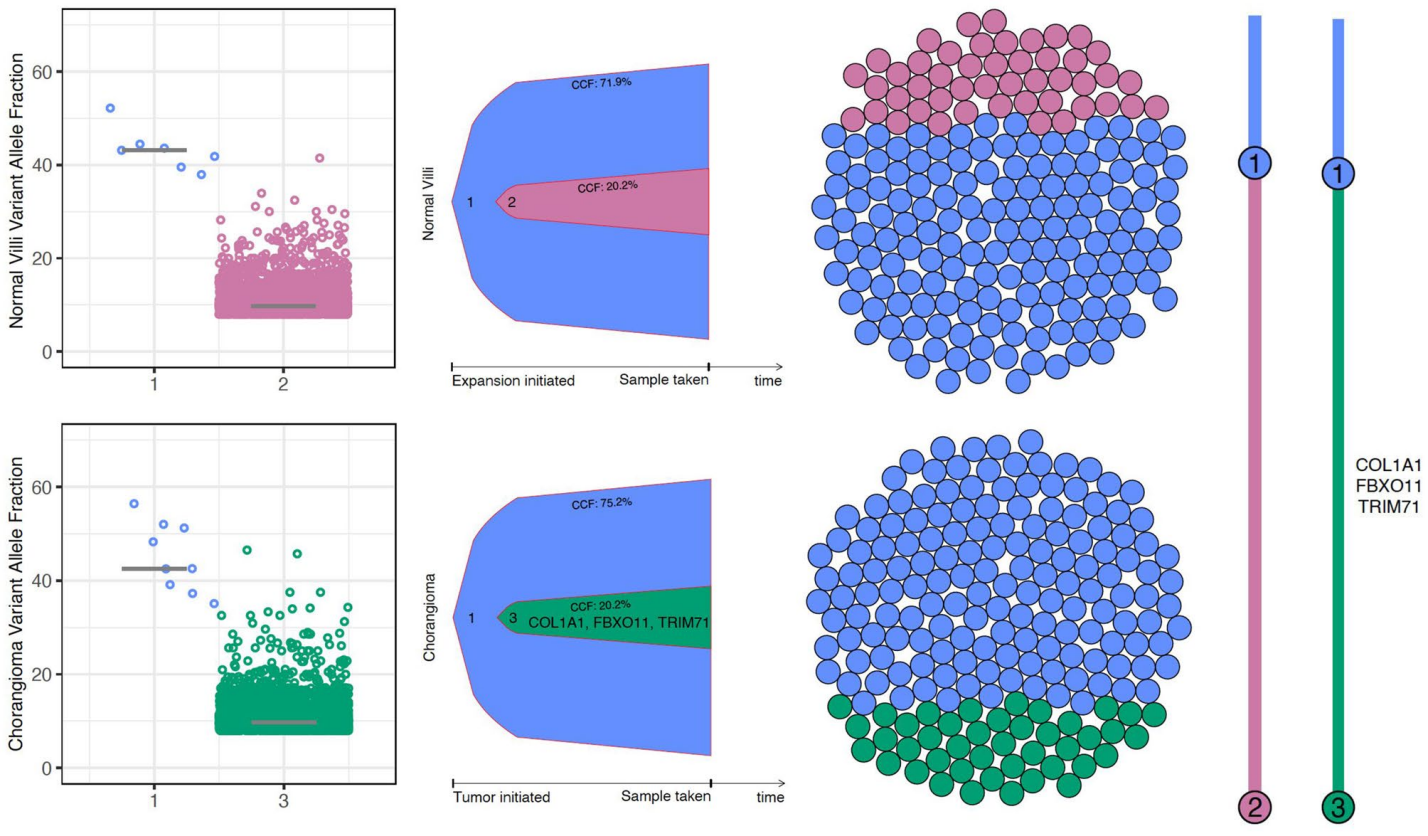
Chorangioma clonal evolution. Scatter plots show the cancer cell fraction (CCF) of each clonal cluster. Bell plots represent clonal dynamics over time and the sphere of cells depict the estimated subpopulation fractions. The branching clonal evolutionary model (far right) shows the predicted clonal evolution of chorangioma tissue and normal villi. It is clear from this model that cluster 3 indicates a subclonal population in the chorangioma not seen in the normal villi

We hypothesized that patterns of somatic variation patterns in these chorangiomas might resemble those in malignancy, where development of subclones and evolving subclonal architecture can reveal mutational processes. Clonal analysis identified a linear model of molecular evolution, showing expansion of a subclonal population (population 3) exclusively in the chorangioma (Fig. 2). These insights, pending experimental confirmation, support the hypothesis that mutational processes may operate in these tissues before or during chorangioma development.

## Mutational signatures of chorangiomas

**Fig. 3:**
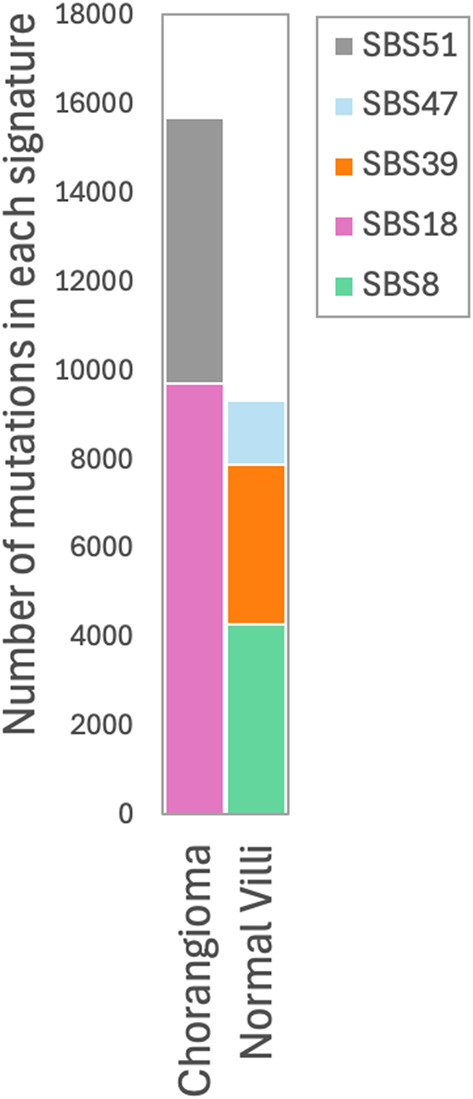
Mutational signature analysis. The majority of somatic variation identified in the chorangioma sample was attributed to the ROS induced SBS18 signature. An equal amount of variation in the normal villi was attributed to SBS8 and SBS39 signatures. The chorangioma and to a lesser degree the normal villi sample also had patterns of somatic variants attributable to likely sequencing artifacts (SBS47 and SBS51)

Somatic variation can result from events that leave distinct mutational signatures reflecting underlying processes, such as replication errors, repair deficiencies, environmental exposures, and oxidative stress. Application of these methods identified a dominant signature, SBS18, in baby A’s chorangioma sample, (Fig. 3, Supplemental Fig. 2), which has been associated with DNA damage from reactive oxygen species (ROS) [116, 117]. While we hypothesize that the presence of this ROS-associated mutational signature SBS18 suggests oxidative stress involvement perhaps due to rapid cell proliferation, high metabolic activity, or abnormal vasculature with frequent hypoxia-reperfusion cycles not present in normal placenta [118–120] definitive evidence, such as direct ROS measurements (e.g. 4-HNE staining), could not be generated due to sample constraints. Thus, we interpret these findings cautiously, emphasizing the need for validation through future research. SBS18 has been observed at lower levels in healthy placental tissue [48]. SBS51, also present in this sample, is linked to sequencing artifacts [50, 121].

SBS8 is the dominant mutational signature seen in the normal villous tissue from baby B’s placental territory. It is associated with deficiencies in homologous recombination/nucleotide excision repair (HR/NER) [122, 123] and is previously unreported in placental tissue. The placenta’s rapid growth and physiological stress may increase DNA damage, activating alternative repair pathways. If so, we hypothesize that SBS8 may reflect high demand on repair systems during normal development, rather than pathology. SBS39 was also identified. This signature has been linked to DNA polymerase eta (POLH) A > T mutations during DNA replication under UV exposure, and noted in embryonal rhabdomyosarcoma, but the association here is unclear [124]. SBS47, a minor component associated with sequencing artifacts [50], was observed at low levels, and was less prominent in normal villi than in chorangioma tissue, likely due to differences in DNA quality between tissues.

## Discussion

**Fig. 4:**
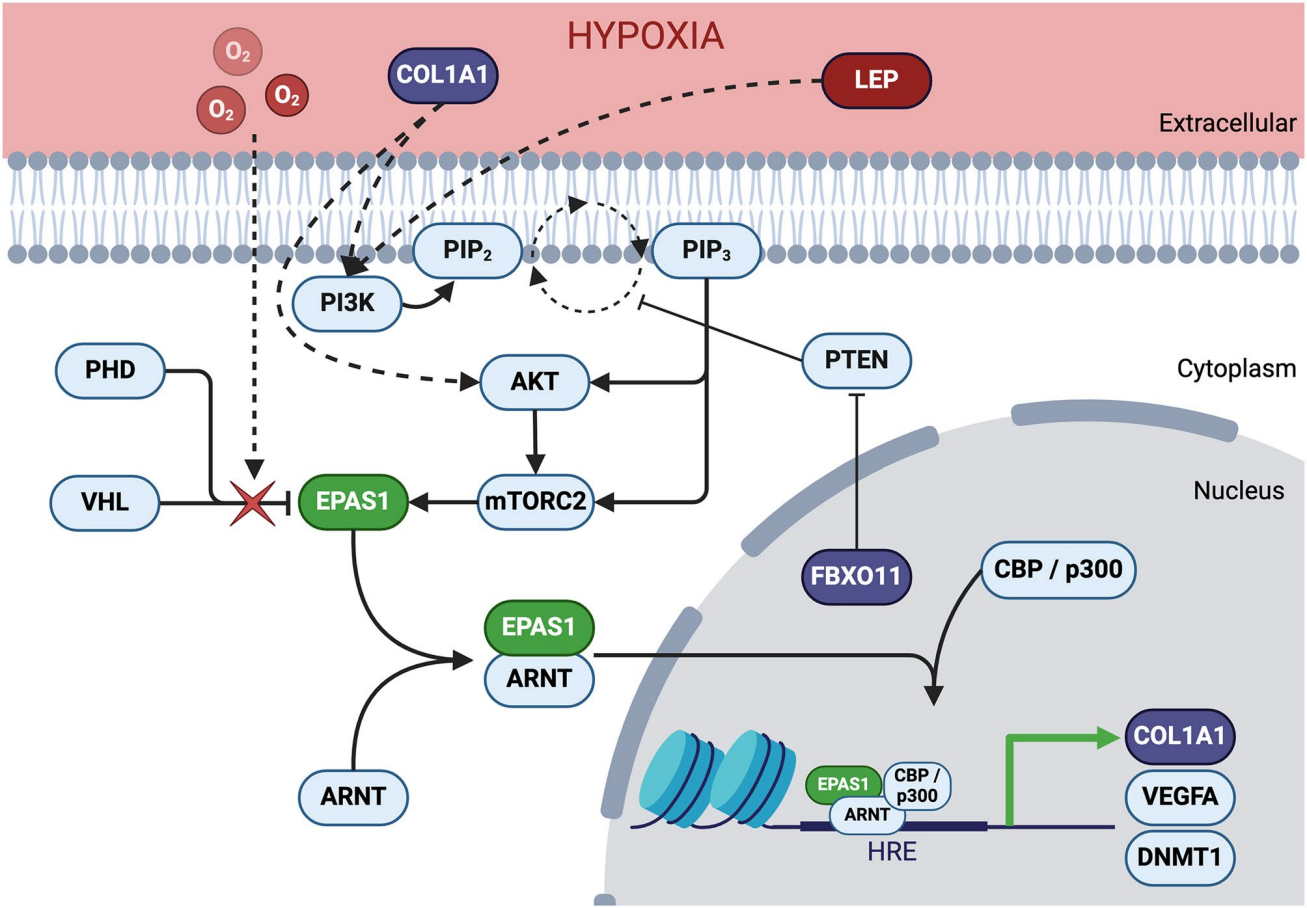
Hypoxia driven EPAS1 stabilization. Under normoxia, PHD hydroxylates EPAS1 (in green) allowing VHL to ubiquitinate EPAS1 leading to proteasomal degradation. Under hypoxia, PHD fails to hydroxylate EPAS, preventing VHL binding, leading to EPAS1 accumulation. This triggers EPAS1 dimerization with ARNT, nuclear translocation, recruitment of CBP/p300, and activation of target genes containing hypoxia response elements in their promoter. Accumulation of ROS increases somatic mutation in genes within the chorangioma (in purple). These ROS driven alterations are identified as ROS damage and deficient repair mutational signatures and contribute to abnormal capillary proliferation and genomic instability, leading to altered placental growth dynamics. Changes in expression (in red) also impact angiogenesis, vascularization, and placental integrity. We hypothesis that the combination of EPAS1 variation, MCDA twin pregnancy, and COL1A1, FBXO11, and TRIM71 somatic mutation in the baby A’s larger placental territory impacted placental development, blood vessel formation and tissue integrity, and angiogenesis, ultimately resulting in the abnormal vascular structures of the chorangiomas in that placental share only

Placental development is a carefully coordinated process influenced by oxygen levels, which play a crucial role in placentogenesis by regulating villous vascularization, and trophoblast differentiation and proliferation. Chronic environmental hypoxia and certain molecular or genetic changes have been suggested to drive aberrant angiogenesis and chorangioma formation [3, 7–13], though definitive evidence has been limited. Our findings from a unique case of multiple chorangiomas in half of a shared placenta of monozygotic MCDA twins suggest a multi-step pathology leading to chorangioma development (Fig. 4).

We identified a heterozygous frameshift deletion in *EPAS1* across all sequenced tissues, suggesting early embryonic or germline origin during pre-morula development (days 1–3). We hypothesize that this variant blunts placental ability to sense and respond to the low-oxygen environment of early pregnancy, where *EPAS1* typically stabilizes to support development. We further propose that this impairment would be further exacerbated in this case by subsequent formation of MCDA twins sharing a placenta (days 4–8), resulting in increased susceptibility to oxidative stress and damage, given the elevated resource demands and limited adaptive capacity inherent to MCDA pregnancies. We propose that this combination of early first-trimester localized hypoxia due to MCDA twinning and *EPAS1* alteration would be likely to increase reactive oxygen species and subsequent DNA damage.

In affected baby A’s placental territory, we identified pathogenic and likely pathogenic somatic mutations in *TRIM71*, *FBXO11*, and *COL1A1.* Based on their known biological functions in angiogenesis, cell-cycle regulation, oxidative stress responses, and extracellular matrix integrity, we suggest that these variants may contribute to localized abnormal vascular proliferation and disrupted hypoxia-responsive pathways, influencing chorangioma formation. Although our observations point to potential parallels between somatic variation patterns in chorangiomas and malignancies, no direct evidence currently confirms that chorangiomas share cancer-like mutational processes. Therefore, our interpretations remain speculative, pending further investigation. Additional factors, such as asymmetrical placental territories and maternal oxygenation, likely also influenced chorangioma development.

While our findings are novel and point to potential mechanisms of disease, we acknowledge a number of limitations. RNA and DNA degradation from FFPE likely limited certain aspects of our expression analysis and mutational signature profiling [33]. Analysis from fresh or frozen tissues (which was not possible in this case) would have been preferable. Although an optimized pipeline was used for clonal evolution [125], identification of lower VAF subclonal populations was limited by WGS depth and single tissue sampling [126]. Statistical interpretation in single-case studies remains challenging, as rare variants in low-prevalence conditions rarely meet traditional significance thresholds. Additional research is needed to determine whether *EPAS1* involvement is specific to this case or indicative of broader patterns in multiple or large chorangiomas. The rarity of multiple chorangiomas will make gathering additional samples challenging. Further investigation, including functional validation via CRISPR/Cas9 editing in trophoblast or endothelial cells, single-cell analyses at maternal-placental interfaces, or spatial transcriptomics, would help clarify the potential hypoxic response, angiogenesis, and DNA repair impacts of the somatic variants identified. Modeling these alterations in systems that closely resemble human placental physiology, such as guinea pigs [127] or baboons [128], would enhance our understanding of EPAS1 haploinsufficiency and hypoxia-related effects, confirming the role of these variants in chorangioma formation and expanding our understanding of placental pathophysiology. We therefore interpret our findings with caution, acknowledging that the observed *EPAS1* variant and molecular signatures may represent just one of multiple possible pathways to chorangioma formation.

In summary, we present molecular findings from a unique case of multiple chorangioma syndrome in monozygotic MCDA twins, affecting only one twin’s placental territory. To our knowledge, this is the first report to implicate a molecular mechanism in multiple chorangioma syndrome and the first to propose the interaction of germline and somatic variants in the pathobiology of these tumors. While chorangiomas are typically benign, our analysis suggests there may be mutational processes with potential oncogenic activity involved, but we interpret these findings cautiously, emphasizing the need for validation through future research. This case underscores the potential for complex genetic and environmental interplay in chorangioma formation, advancing our understanding of placental biology and suggesting directions for future investigations on the interplay between germline, somatic, and environmental factors as similar cases emerge.

## Electronic supplementary material

Below is the link to the electronic supplementary material.

Supplementary material 1

Supplementary material 2

## Data Availability

Somatic variants, code, and documentation on the mutational signature analysis can be found in our GitHub repository athttps://github.com/uab-cgds-worthey/somatic-mutation-signal-identification. The input somatic variants, CNVKit genome-wide copy-number estimates, input merging and running instructions for variant clustering using PyClone-VI and clonal evolution by ClonEvol are available in our GitHub repository athttps://github.com/uab-cgds-worthey/multiple-chorangiomas-subclone-analysis. The principal author takes full responsibility for the data presented in this study, analysis of the data, conclusions, and conduct of the research. Datasets generated from the analysis presented will be available from the corresponding author on reasonable request.

## Abbreviations

ACMG: American College of Medical Genetics and Genomics
AMP: Association for Molecular Pathology
ARNT: Aryl Hydrocarbon Receptor Nuclear Translocator
BER: Base excision repair
BWS: Beckwith-Wiedemann syndrome
CBP: CREB-binding protein
CCF: Cancer cell fraction
CDKN1A/p21: Cyclin Dependent Kinase Inhibitor 1A
COL1A1: Collagen type I alpha 1 chain
COSMIC: Catalogue Of Somatic Mutations In Cancer
CREBBP: CREB-binding protein
DNMT1: DNA methyltransferase 1
DTL: Denticleless E3 ubiquitin protein ligase homolog
ECM: Extracellular matrix
EP300: E1A binding protein p300
EPAS1: Endothelial PAS domain protein 1
FBXO11: F-box protein 11
FFPE: Formalin-Fixed Paraffin-Embedded
HELLP: Hemolysis, Elevated Liver enzymes and Low Platelets
HIF1A: Hypoxia inducible factor 1 subunit alpha
HIF1B: Hypoxia inducible factor 1 subunit beta
HIF2A: Hypoxia inducible factor 2 subunit alpha
HIF3A: Hypoxia inducible factor 3 subunit alpha
HRE: Hypoxia response element
IUGR: Intrauterine growth restriction
LEP: Leptin
MCDA: Monochorionic, diamniotic
MUTYH: MUTY DNA glycosylase
NMD: Nonsense-mediated mRNA decay
OMIM: Online Mendelian Inheritance in Man
PHD: Hypoxia-inducible factor prolyl hydroxylase
PI3K: Phosphoinositide 3-kinases
PI3K-AKT: Phosphoinositide 3-kinases-Akt serine/threonine kinase pathway
POLH: DNA polymerase eta
PTEN: Phosphatase and tensin homolog
RNA-Seq: Bulk RNA sequencing
ROS: Rreactive oxygen species
SBS: Single base substitution
SNV: Single nucleotide variant
TRIM71: Tripartite motif containing 71
VAF: Variant allele fraction
VEGF: Vascular endothelial growth factor
VHL: Von Hippel-Lindau tumor suppressor
VUS: Variant of unknown significance
WGS: Whole genome sequencing
