# Supplementary figures and images for: Multi-omic analysis identifies a multi-step pathology in a case of multiple chorangioma syndrome in monochorionic twins
Source: Orphanet J Rare Dis. 2026 Feb 3;21:56. doi: 10.1186/s13023-026-04228-2 (PMC12895669; doi:10.1186/s13023-026-04228-2)

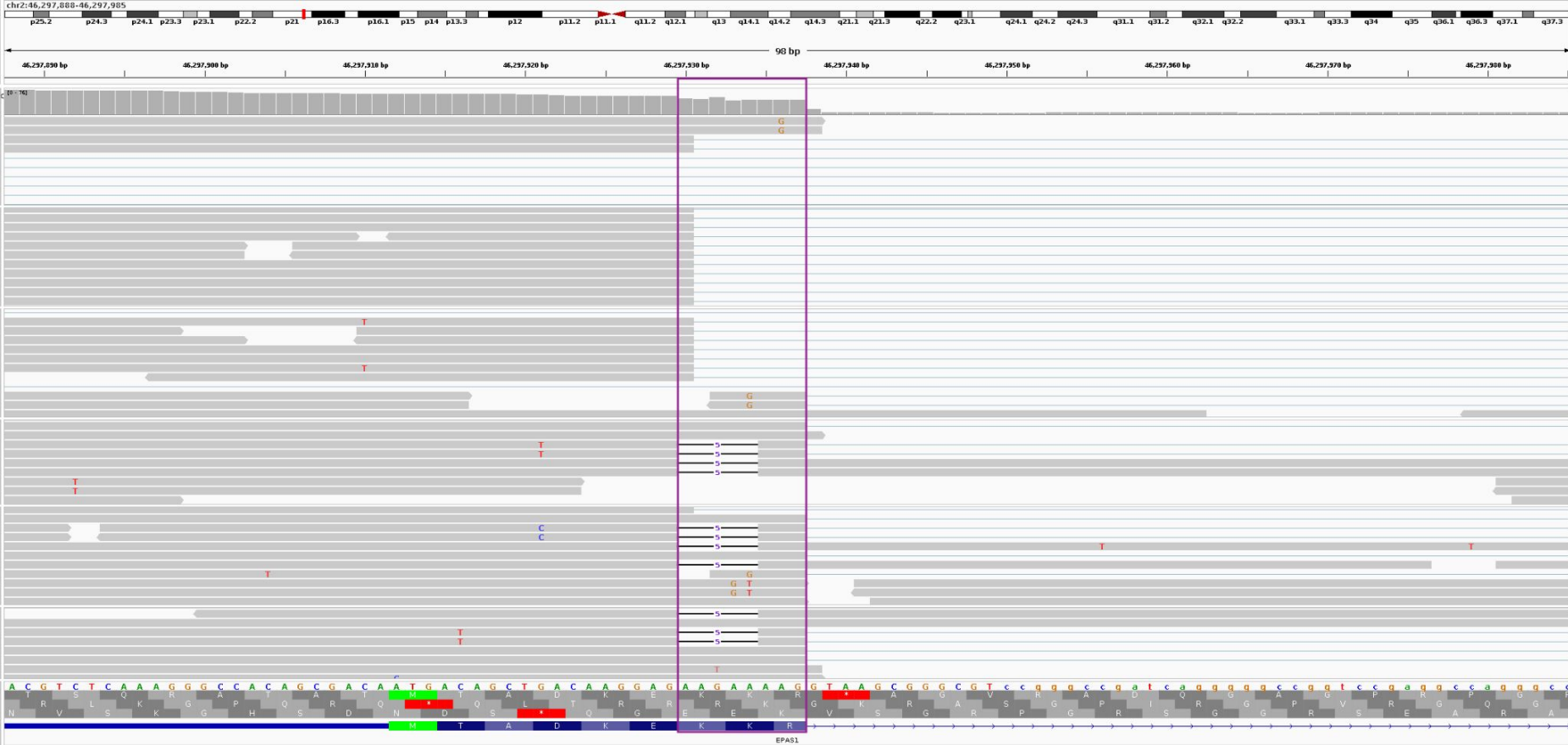

Supplement: Supplementary file 1 — Supplementary material 1 [file 13023_2026_4228_MOESM1_ESM.pdf]

A

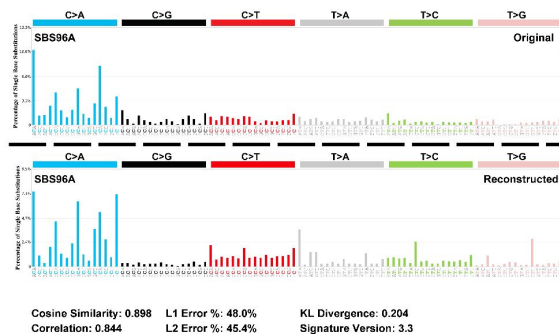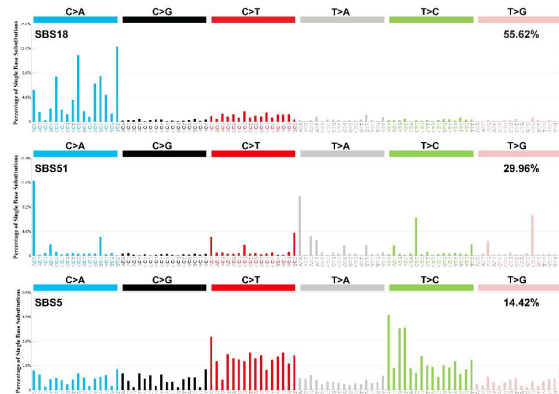

B

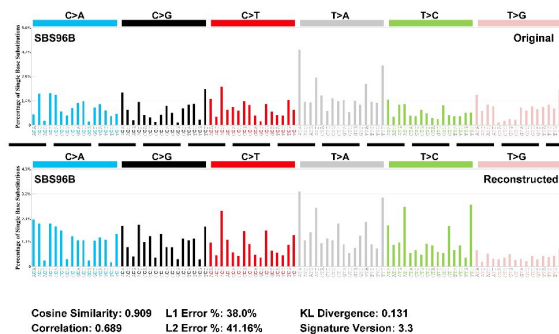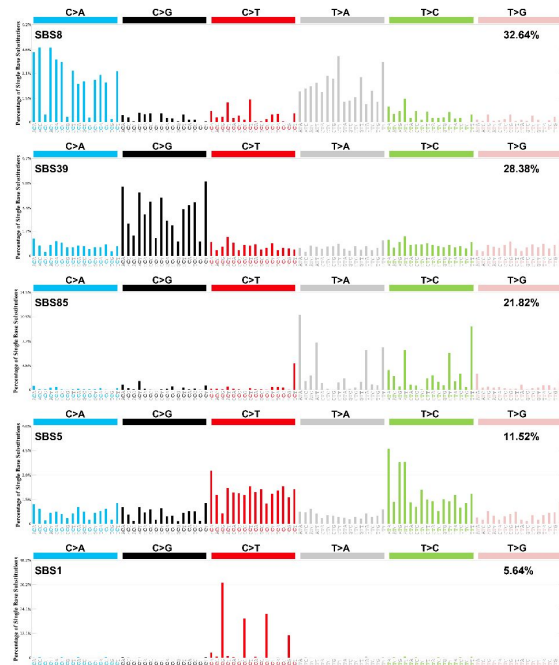

Supplement: Supplementary file 2 — Supplementary material 2 [file 13023_2026_4228_MOESM2_ESM.pdf]
